# Update on the Roles of Polyamines in Fleshy Fruit Ripening, Senescence, and Quality

**DOI:** 10.3389/fpls.2021.610313

**Published:** 2021-02-10

**Authors:** Fan Gao, Xurong Mei, Yuzhong Li, Jiaxuan Guo, Yuanyue Shen

**Affiliations:** ^1^Key Laboratory for Northern Urban Agriculture of Ministry of Agriculture and Rural Affairs, Department of Resources and Environment, Beijing University of Agriculture, Beijing, China; ^2^Water Resources and Dryland Farming Laboratory, Institute of Agricultural Environment and Sustainable Development, Chinese Academy of Agricultural Sciences, Beijing, China

**Keywords:** polyamines, fruit ripening, ABA, ethylene, NO, Ca^2+^, review

## Abstract

Ripening of fleshy fruits involves complex physiological, biochemical, and molecular processes that coincide with various changes of the fruit, including texture, color, flavor, and aroma. The processes of ripening are controlled by ethylene in climacteric fruits and abscisic acid (ABA) in non-climacteric fruits. Increasing evidence is also uncovering an essential role for polyamines (PAs) in fruit ripening, especially in climacteric fruits. However, until recently breakthroughs have been made in understanding PA roles in the ripening of non-climacteric fruits. In this review, we compare the mechanisms underlying PA biosynthesis, metabolism, and action during ripening in climacteric and non-climacteric fruits at the physiological and molecular levels. The PA putrescine (Put) has a role opposite to that of spermidine/spermine (Spd/Spm) in cellular metabolism. Arginine decarboxylase (ADC) is crucial to Put biosynthesis in both climacteric and non-climacteric fruits. *S*-adenosylmethionine decarboxylase (SAMDC) catalyzes the conversion of Put to Spd/Spm, which marks a metabolic transition that is concomitant with the onset of fruit ripening, induced by Spd in climacteric fruits and by Spm in non-climacteric fruits. Once PA catabolism is activated by polyamine oxidase (PAO), fruit ripening and senescence are facilitated by the coordination of mechanisms that involve PAs, hydrogen peroxide (H_2_O_2_), ABA, ethylene, nitric oxide (NO), and calcium ions (Ca^2+^). Notably, a signal derived from PAO5-mediated PA metabolism has recently been identified in strawberry, a model system for non-climacteric fruits, providing a deeper understanding of the regulatory roles played by PAs in fleshy fruit ripening.

## Introduction

Fleshy fruits are rich in sugars, vitamins, minerals, antioxidants, and fibers, which are essential components of food and contribute to human nutrition and health. Fruits ripen *via* a cascade of complex physiological, biochemical, and molecular processes that coincide with changes in the texture, color, flavor, and aroma of fruits. Based on their respiration rate, ethylene emissions, and ripening process, fleshy fruits can be classified into climacteric and non-climacteric types. In climacteric fruits, such as the prototype model species tomato (*Solanum lycopersicum*), ripening is controlled by ethylene (Alexander and Grierson, 2002). In non-climacteric fruits, such as strawberry (*Fragaria ananassa*), ripening is largely controlled by ABA (Li et al., 2011; Shen and Rose, 2014). In addition to ABA and ethylene, a series of studies also suggested a critical role for PAs in fruit ripening (Dibble et al., 1988; Kushad et al., 1988; Rodriguez et al., 1999; Lester, 2000; Bregoli et al., 2002; de Dios et al., 2006; Mattoo et al., 2006, 2007, 2010; Handa and Mattoo, 2010; Parra-Lobato and Gomez-Jimenez, 2011; Koushesh saba et al., 2012; Gupta et al., 2013; Fortes et al., 2015; Jiang et al., 2015; Simpson et al., 2017; Guo et al., 2018; Liu et al., 2018; Shukla et al., 2020). In the past several years, many reviews have explored the roles of ABA and ethylene in the ripening of fleshy fruits, while a handful of reviews have reported on the roles of PAs in response to development and stress in plants (Kusano et al., 2018; Chen et al., 2019; Fortes et al., 2019; Wang et al., 2019; Killiny and Nehela, 2020). However, far fewer reviews have focused on the role of PAs in the ripening process (Mattoo and Handa, 2008; Fortes and Agudelo-Romero, 2018). Here, we will review the current knowledge regarding the biosynthesis, metabolism, and function of PAs in the ripening and senescence of fleshy fruits, by systematically comparing climacteric and non-climacteric species.

## Roles of PAs in the Ripening, Senescence, and Quality of Fleshy Fruits

PAs are aliphatic amines present in all living organisms and have been extensively studied, including in the context of plant growth and morphogenesis, as well as fruit development (Bregoli et al., 2002; Khan and Singh, 2010; Kolotilin et al., 2011; Torrigiani et al., 2012; Teh et al., 2014; Fatima et al., 2016; Sharma et al., 2017; Fortes and Agudelo-Romero, 2018; Anwar et al., 2019). In agreement with an early report that stated “no growth, no PA production” (Russell, 1973), it is now widely accepted that polyamines (PAs), mainly putrescine (Put), spermidine (Spd), and spermine (Spm), play important roles in diverse biological processes and stress responses in plants (Handa and Mattoo, 2010; Mattoo et al., 2010; Moschou et al., 2012; Kusano et al., 2018; Fortes et al., 2019; Wang et al., 2019).

In the past decades, much progress has been made toward a better understanding of the roles of PAs in various fleshy fruits, such as avocado (*Persea americana*; Kushad et al., 1988), tomato (Dibble et al., 1988; Mehta et al., 2002; Mattoo et al., 2006, 2007, 2010), eggplant (*Solanum melongena*; Rodriguez et al., 1999), muskmelon (*Cucumis melo*; Lester, 2000), peach (*Prunus persica*; Bregoli et al., 2002), damson plum (*Prunus salicina*; de Dios et al., 2006), olive (*Canarium album*; Parra-Lobato and Gomez-Jimenez, 2011), apricot (*Prunus armeniaca*; Koushesh saba et al., 2012), apple (*Malus sylvestris var. domestica*; Kitashiba et al., 2005), loquat (*Dimocarpus longgana* Lour; Jiang et al., 2015), grape (*Vitis vinifera*; Fortes et al., 2015), raspberry (*Rubus idaeus*; Simpson et al., 2017), and strawberry (Guo et al., 2018; Mo et al., 2020).

### The Roles of PAs in Climacteric Fruits

The roles of PAs have been extensively studied during tomato fruit development and ripening (Mutschler, 1984; Dibble et al., 1988; Saftner and Baldi, 1990; Bouchereau et al., 1999; Mehta et al., 2002; Mattoo et al., 2006, 2007; Tassoni et al., 2006; Gapper et al., 2013; Tsaniklidis et al., 2016; Sharma et al., 2017; Hao et al., 2018; Liu et al., 2018). PA levels appear to vary between cultivars (cv), as Put, Spd, and Spm contents decrease during fruit ripening in cultivars Pik Red and Rutgers, whereas fruits from the cv Liberty ripen slowly, have a prolonged keeping quality, and are characterized by a high accumulation of Put during ripening (Dibble et al., 1988; Saftner and Baldi, 1990). Similarly, in cherry tomato (*Solanum lycopersicum* cv *cerasiforme*) fruits, Put contents increase gradually during maturation and reach their peak at the red ripe stage, while the levels of Spd and Spm decrease during the ripening process (Tsaniklidis et al., 2016). PAs are not thought to be directly involved in delaying tomato fruit ripening, but rather may limit the rate of ripening or over-ripening (Tassoni et al., 2006). Expressing the yeast (*Saccharomyces cerevisiae*) *S-adenosylmethionine decarboxylase* (*SAMDC*) gene specifically in tomato fruits results in the ripening-specific accumulation of lycopene and sugars, as well as Spd and Spm, which are normally low in the nontransgenic tomato parent (Mehta et al., 2002). Further work determined that the pathways involved in nitrogen sensing/signaling and carbon metabolism are preferentially activated in these transgenic fruits with high Spd/Spm contents when compared to nontransgenic fruits (Mattoo et al., 2006). Indeed, Spd/Spm may act as an anabolic growth regulator that reflects the contents of organic nitrogenous metabolites in fruit cells, eventually leading to the improvement of fruit quality once Spd and Spm levels have reached a critical threshold (Mattoo et al., 2007; Srivastava et al., 2007). These results, to a large extent, indicate that PAs act as an activating signal for a vast genetic network involved in the regulation of tomato fruit growth, development, and senescence (Mattoo and Handa, 2008). Notably, transcriptome and metabolome profiling from three genotypes (one wild type, two transgenic lines carried with the yeast *SAMDC*) at two stages of ripening (pink and red) established that the juice parameters including soluble solids, acidity, viscosity, lycopene content, and ethylene production were positively associated with Spd and Spm levels but anti-correlated with Put levels (Handa and Mattoo, 2010). Put content in the pericarp tissue of tomato fruits from the control varied little during ripening, whereas Put content decreased several-fold and Spd/Spm levels drastically increased during ripening in the transgenic lines (Mattoo et al., 2010). In addition, transgenic tomato plants overexpressing the yeast spermidine synthase (SPDS) gene accumulate a high level of Spd in fruit, resulting in a delay of ripening onset and longer shelf life. These results, therefore, suggest that Put is predominately associated with catabolic processes, while Spd and Spm contents are positively correlated with anabolic processes (Mattoo et al., 2010; Nambeesan et al., 2010). The importance of the PA Put in extending tomato fruit shelf life has recently come into focus, together with Spd and Spm (Gupta et al., 2019; Osorio et al., 2020). Notably, Spd/Spm may regulate small nucleolar RNAs (snoRNAs) and ribosomal RNA (rRNA) expression directly or indirectly, which in turn will affect protein biosynthesis, metabolism, and other cellular activities in a positive manner (Shukla et al., 2020).

Outside of the tomato literature, roles for PAs have also been reported during ripening for other climacteric fruits. For example, in peach fruit, the contents of Spd and Spm gradually decrease until harvest, while Put levels exhibit a peak just before the onset of ethylene production, reaching a second peak at harvest time (Liu et al., 2006). Similarly, Spd and Spm decrease while Put increases in the pulp during the ripening of banana (*Musa paradisiaca*) fruit (Borges et al., 2019). In honeydew melon (*Cucumis melo*), the ratio between endogenous Spd and Put changes about 5 days before harvest, from Spd > Put to Spd < Put, concomitantly with the onset of fruit senescence, demonstrating that high endogenous Spd contents may delay melon senescence, promote fruit lycopene accumulation, and extend shelf life (Lester, 2000; Neily et al., 2011). In apple, Spd was reported to be the predominant form of PAs during fruit development and ripening (Zhang et al., 2003). Interestingly, PAs have both short- and long-term effects on peach fruit ripening (Torrigiani et al., 2012).

Collectively, Put levels increase during conditions of low metabolic activity, while Spd and Spm act as growth and ripening stimulators. Higher PA levels also contribute to maintaining cellular vitality and longer vine life for ripening tomato fruits. To some extent, the Spd/Put ratio controls ripening, senescence, and quality in climacteric fruits. PAs serve not just as nitrogen-rich compounds but also as signaling molecules with specific biological functions when Spd and Spm reach a critical threshold.

### The Roles of PAs in Non-climacteric Fruits

In comparison to the many advances describing the roles of PAs in climacteric fruits over many years, it is only recently that breakthroughs as in grape and strawberry have been made toward elucidating whether and how PAs participate in non-climacteric fruit ripening, senescence, and quality (Agudelo-Romero et al., 2013, 2014; Fortes et al., 2015; Guo et al., 2018; Mo et al., 2020).

During grape fruit ripening, Put and Spd contents decrease sharply, while Spm contents remain constant (Agudelo-Romero et al., 2013). Similarly, grape berry development is accompanied by a gradual decrease in PA levels, indicating the important role played by PA catabolism in ripening (Agudelo-Romero et al., 2014). Using strawberry as a model for non-climacteric fruits, Spm contents rise strongly after the fruit starts turning red, and Spm is a dominant component of the ripe fruit. Moreover, treatment with exogenous Put inhibits ripening, in contrast to the acceleration of ripening caused by exogenous Spd or Spm, thus confirming a crucial role for Spm in the regulation of strawberry fruit ripening (Guo et al., 2018). Additionally, in orange (*Citrus sinensis*) fruit, Put contents increase in the peel and flesh during ripening and peak at the ripe stage (Tassoni et al., 2004).

Taken together, PAs play important roles during the ripening of both climacteric and non-climacteric fruits. PA composition, contents, and actions vary between fruit types and developmental stages. Put appears to function in a manner opposite to that of Spd and Spm, which exhibit similar functions in cellular metabolism. High Put content inhibits fruit ripening and plays a vital role in extending fruit shelf life. By contrast, elevated contents of Spd and Spm promote fruit ripening, a role of Spd in climacteric fruits and Spm in non-climacteric fruits. A metabolic transition toward the conversion of Put to Spd/Spm represents a sign for the onset of fruit ripening.

## PA Biosynthesis and Metabolism in Fleshy Fruits

PA biosynthesis has been described in microorganisms, mammalian cells, and plants (Bachrach, 1970; Kushad et al., 1983). The first PA biosynthesis enzymes whose activity was measured in fleshy fruits were ornithine decarboxylase in tomato (Cohen et al., 1982) and arginine decarboxylase in avocado (Winer et al., 1984). Much progress has been made toward elucidating the PA biosynthetic pathway and PA metabolism in climacteric fruits, largely using tomato as a model (Kushad et al., 1988; Rastogi and Davies, 1990, 1991; Mehta et al., 2002; Nambeesan et al., 2010; Kolotilin et al., 2011; Neily et al., 2011; Pandey et al., 2015; Tsaniklidis et al., 2016; Liu et al., 2018). Encouragingly, the biosynthesis and metabolism of PAs in non-climacteric fruits have also been recently deciphered, using strawberry as the model plant (Guo et al., 2018; Mo et al., 2020).

### PA Biosynthesis and Metabolism in Climacteric Fruits

Several enzymes have been linked to PA biosynthesis and metabolism in climacteric fruits: arginine decarboxylase (ADC), ornithine decarboxylase (ODC), spermidine synthase (SPDS), spermine synthase (SPMS), copper amine oxidase/diamine oxidase (CuAO/DAO), and polyamine oxidase (PAO) (Fortes and Agudelo-Romero, 2018). In this section, we focus on the roles of key genes and enzymes related to PA metabolism during ripening, largely using tomato as a model.

Polyamines are synthesized from arginine and ornithine (Lasanajak et al., 2014). During the early stages of fruit development, ODC activity predominates over that of ADC, but ADC activity becomes more prominent in the later stages (Cohen et al., 1982; Teitel et al., 1985). The increase in Put content in ripe fruit parallels the increase in ADC activity, indicating that ADC activity determines Put levels (Rastogi and Davies, 1990, 1991). Notably, an early report detected PAO activity in tomato pericarp tissues; PAOs catalyze the conversion of Spm to Spd and Put (Rastogi and Davies, 1990). Tsaniklidis et al. (2016) observed that *CuAO* and *SPMS* were mostly expressed during rapid fruit growth, with a high expression level for *CuAO* during mature green and breaker stages contributed to tomato fruit ripening, and *SPDS1* expression peaked at the red ripe stage. Compared to the minimal expression detected for *SlSPMS*, *SlADC*, and *SlODC* during tomato fruit ripening, *SlSPDS2* may play a prominent role in ripening (Liu et al., 2018), which would agree with the important roles assigned to the SAMDC and SPDS enzymes in the control of Spd biosynthesis (Kolotilin et al., 2011; Neily et al., 2011; Pandey et al., 2015; Liu et al., 2018). Overexpression of *SAMDC*, *SPDS*, or *ODC* leads to an increase in PA contents, which improves fruit quality and prolongs shelf life (Mehta et al., 2002; Mattoo et al., 2007; Nambeesan et al., 2010; Neily et al., 2011; Pandey et al., 2015). Thus, ADC, SAMDC, and SPDS play vital roles in the regulation of tomato fruit ripening, with ADC specifically contributing to high Put content and SAMDC and SPDS contributing to elevated Spd content.

Aside from tomato, PA biosynthesis and metabolism have been characterized to some extent during fruit development of other climacteric fruits. ODC activity was low in avocado during flowering but was followed by a threefold increase during early fruit development (within 60 days after flowering) and then a decrease in the late stage of ripening; ADC activity showed a minor increase over the entire period (Kushad et al., 1988). In peach, *ADC* transcript levels remained constant during fruit ripening, while *ODC* expression first rose sharply before decreasing at late stages. However, the transcripts levels of *ADC*, *ODC*, *SAMDC*, *SPDS*, and *SPMS* did not correlate with enzyme activity between pre-harvest and post-harvest stages (Ziosi et al., 2003; Liu et al., 2006), suggesting that PA biosynthesis and metabolism in pulp cells are regulated at both the transcriptional and translational levels during fruit ripening and senescence.

### PA Biosynthesis and Metabolism in Non-climacteric Fruits

Polyamine biosynthesis and metabolism also contribute to non-climacteric fruit ripening, as shown in grape and strawberry (Deluc et al., 2007; Fortes et al., 2011; Agudelo-Romero et al., 2013, 2014; Guo et al., 2018; Mo et al., 2020). The development of grape berries goes through two successive periods of sigmoidal growth separated by a lag phase known as veraison, marking the onset of ripening, or the transition from de-greening to coloration (Coombe and McCarthy, 2000). The expression levels of grape *ADC*, *SPDS*, and *SPMS* rise at the onset of ripening and remain high in ripe fruits (Deluc et al., 2007). An increase in the content of gamma-aminobutyric acid (GABA) during grape ripening suggests that PA oxidation leads to a decrease in PA levels (Fortes et al., 2011). Manipulation of PA catabolism using the exogenous application of guazatine (an inhibitor of polyamine oxidase; Maiale et al., 2008) regulates grape ripening by inhibiting PAO activity (Agudelo-Romero et al., 2014). During grape berry ripening, levels for free and conjugated PAs drop drastically, even though the expression of *ADC* is up-regulated and the enzymatic activity of CuAO/DAO and PAO increases, as well as H_2_O_2_ content, suggesting a vital role for PA catabolism in grape berry ripening (Agudelo-Romero et al., 2013). Nevertheless, ODC plays a role in the biosynthesis of PAs in grape, in the context of the concomitant downregulation of *SPDS* expression and the upregulation of *SAMDC1* during grape berry ripening (Agudelo-Romero et al., 2013). Together, PA metabolism plays a pivotal role in grapevine fruit ripening.

In strawberry, SAMDC has high enzymatic activity, with a *K*_*d*_ of 170 μM. The manipulation of *FaSAMDC* expression levels modulates fruit ripening, demonstrating that FaSAMDC plays an important role in ripening (Guo et al., 2018). Similarly, the expression of a raspberry *SAMDC* gene is induced at the white/red phase during ripening (Simpson et al., 2017). A recent report provided multiple lines of evidence supporting a role for strawberry PAO5, a polyamine oxidase localizing to the cytoplasm and nucleus, in the negative regulation of strawberry fruit ripening. First, *PAO5* transcript and PAO5 protein levels gradually decrease during the ripening process. Second, lowering *PAO5* expression by RNAi promotes the accumulation of Spd and Spm and fruit ripening. Third, *FaPAO5* plays a role in the terminal catabolism of Spd and Spm, with a *K*_*d*_ of 0.21 μM for Spd and 0.29 μM for Spm (Mo et al., 2020).

Collectively, ADC is a crucial enzyme for Put production in climacteric and non-climacteric fruits. Generally, Spd biosynthesis catalyzed by the enzymes SAMDC and SPDS is critical to ripening in climacteric fruits, while Spm biosynthesis mediated by SAMDC and SPMS fulfills a similar role in non-climacteric fruit ripening. PAO-mediated PA metabolism, directly or indirectly as a signal, regulates PA homeostasis and fruit ripening, as was shown in strawberry.

## Mechanisms of PA Action in Ripening, Senescence, and Quality of Fleshy Fruits

The key substrate for PA biosynthesis is methionine, which is also used for both PA and ethylene biosynthesis as well as methylation reactions (Mehta et al., 2002; Mattoo and Handa, 2008; Sobolev et al., 2014; Fortes and Agudelo-Romero, 2018). In plant cells, PA levels are rather high and reach molar concentrations; thus, PAs also serve as a storage pool for nitrogen. PA metabolism is connected to the tricarboxylic acid (TCA) cycle, glycolysis, the urea cycle, GABA, nitric oxide (NO), glutamate, 3-alanine, proline, and H_2_O_2_ (Mattoo et al., 2010; Fortes and Agudelo-Romero, 2018). Therefore, PAs play many physiological roles in plants, ranging from nutrition and regulation to signaling, and are linked to a complex metabolic network (Rastogi and Davies, 1990; Mattoo et al., 2006; Neill et al., 2008; Moschou et al., 2012; Lasanajak et al., 2014; Kusano et al., 2018). In this section, we review the relationships between PAs and ABA, H_2_O_2_, NO, and calcium ions (Ca^2+^).

### PAs and Ethylene

*S*-adenosyl-L-methionine (SAM) is a common substrate for the biosynthesis of both PAs and ethylene, thus providing the drive for determining the relationship between PAs and ethylene (Saftner and Baldi, 1990; Mehta et al., 2002; Martínez-Romero et al., 2007; Van de Poel et al., 2013; Sobolev et al., 2014; Pandey et al., 2015).

In tomato, an increase in Put levels in ripe fruits is concomitant with a reduction in climacteric ethylene production (Saftner and Baldi, 1990). Indeed, exogenous application of PAs specifically regulates ethylene biosynthesis by repressing *1-aminocyclopropane-l-carboxylic acid synthase* (*ACC*) expression (Li et al., 1992), while the expression of *SAMDC* is upregulated by ethylene (Van de Poel et al., 2013). The biosyntheses of PA and ethylene occur simultaneously during the ripening of tomato fruits (Mehta et al., 2002), thus placing them in an apparent competition over the same substrate (Gupta et al., 2013). Nonetheless, tomato fruits can produce both ethylene and PAs at high rates during ripening (Van de Poel et al., 2013). The incorporation of ^14^C-Met into Spd and Spm normally declines during tomato fruit ripening, but this is not the case when *SAMDC* is overexpressed, which instead promotes the incorporation of ^14^C-Met into Spd and Spm but not ethylene or ACC during ripening, demonstrating that the cellular flux of SAM is regulated based on fruit demand across competing pathways (Lasanajak et al., 2014). Also, both ethylene-dependent and -independent pathways induce metabolic changes during tomato fruit ripening (Sobolev et al., 2014). Overexpression of *ODC* increases Put, Spd, and Spm contents, which coincides with lower ethylene levels, resulting in delayed on-vine ripening and prolonged shelf life (Pandey et al., 2015).

Similarly, PA and ethylene biosyntheses do not compete for the same pool of substrate during avocado fruit development and ripening (Kushad et al., 1988). PA-dependent ethylene signaling and biosynthesis take place during olive fruit maturation and abscission (Parra-Lobato and Gomez-Jimenez, 2011). In peach, exogenous application of PA strongly inhibits ethylene emissions and delays flesh softening (Bregoli et al., 2002), a stage during which SAM is preferentially transformed into PAs (de Dios et al., 2006). Likewise, an exogenous application of Spd to young developing peach fruits results in the repression of ripening by impairing both ripening-related ethylene and auxin metabolism and signaling (Torrigiani et al., 2012). A delay in ripening induced by jasmonate is also associated with an upregulation of PA levels in peach fruit (Ziosi et al., 2009). In apple, auxin promotes ethylene production, whereas Put, Spd, and Spm repress ethylene production, and this repression is more prominent during the early stages of ripening (Apelbaum et al., 1981).

Taken together, tomato is a powerful model for studying the relationship between PAs and ethylene. In fleshy fruits, this relationship is quite complex. Although SAM is transformed preferentially into PAs at a given condition, the biosynthesis of ethylene and PAs may not always compete for SAM during fruit ripening. Likely due to *de novo* biosynthesis of methionine, the homeostatic regulation of SAM in the face of a higher rate of both ethylene and PA biosynthesis contributes to meeting the complex cellular metabolic requirements for fruit responses to developmental and environmental cues.

### PAs and ABA, NO, H_2_O_2_, and Ca^2+^

Remarkable progress has recently been made linking PAs and other signaling molecules such as H_2_O_2_, NO, and Ca^2+^ in plants (Niu and Liao, 2016; Guo et al., 2018; Toumi et al., 2019; Wang et al., 2019; Mo et al., 2020). Reactive oxygen species (ROS) produced by the action of the PAO enzyme may serve as secondary messengers, whereby the upregulation of *CuAO/PAO* expression further raises ROS levels, resulting in the acceleration of fruit ripening (Wang et al., 2019). Notably, NO and Ca^2+^ act as key signaling molecules linked to H_2_O_2_, which modulates NO and Ca^2+^ signals (Niu and Liao, 2016), suggesting the presence of a complex interactive network among PAs, H_2_O_2_, NO, and Ca^2+^.

For example, high concentrations of Ca^2+^ prevent or partially reverse the effect of PAs during strawberry fruit ripening (Apelbaum et al., 1981). Blocking ethylene biosynthesis similarly affects the levels of both ABA and Put in melon fruits (Concepción Martínez-Madrid et al., 2002). During peach fruit development and ripening, Spd influences the levels of several phytohormones, such as ABA, auxin, gibberellins (GAs), and methyl jasmonate (MeJA) (Ziosi et al., 2009; Kausch et al., 2012; Torrigiani et al., 2012). PAs in general, and Spm in particular, regulate strawberry fruit ripening in an ABA-dominated, IAA-participating, and ethylene-coordinated manner (Guo et al., 2018). At the onset of strawberry fruit ripening, the ABA biosynthetic gene *9-cis-epoxycarotenoid dioxygenase 3* (*NCED3*) is induced, thus promoting the rapid accumulation of ABA, which represses *FaPAO5* expression and further facilitates Spd and Spm accumulation (Mo et al., 2020). The higher levels of Spd and Spm then activate the transcription of *SAMDC*, *SPDS*, and *SPMS*, leading to a positive feedback loop that accelerates Spd and Spm accumulation and ultimately promoting strawberry fruit ripening, suggesting a signaling role for FaPAO5 in ripening, possibly linked to ABA, ethylene, auxin, GAs, and Ca^2+^ (Mo et al., 2020).

Additionally, NO has been reported to be involved in PA-induced metabolism during olive fruit maturation and abscission (Parra-Lobato and Gomez-Jimenez, 2011). In banana, PA biosynthesis *via* an ADC-catalyzed branch is up-regulated several-fold in response to treatment with the NO donor sodium nitroprusside (SNP). NO induces PA biosynthesis *via* the l-arginine-mediated route rather than *via* diversion of the SAM pool, indicating that NO may enhance PA levels *via* ADC during ripening (Lokesh et al., 2019). Taken together, PA catabolism produces H_2_O_2_, which is closely linked to ABA, NO, and Ca^2+^, thereby opening new signaling pathways for exploration into their roles during fruit ripening and senescence.

### PAs and Nitrogen Signaling

It is worth repeating that PA levels in plant cells can reach molar concentrations, making them a potential nitrogen reservoir. PA metabolism is related to multiple signals (Moschou et al., 2012; Niu and Liao, 2016; Guo et al., 2018; Wang et al., 2019). Indeed, high endogenous levels of Spd and Spm exert a marked influence on intermediates of the Krebs cycle, energy molecules (ADP and ATP), and amino acids during tomato fruit ripening (Fatima et al., 2016). Therefore, the Spd/Spm ratio, when reaching a critical minimal threshold, may be perceived as a signaling molecule for nitrogen (N): carbon (C) metabolites by fruit cells during developmental and environmental responses (Mattoo et al., 2006; 2007).

The pathway involved in N sensing/signaling and C metabolism is preferentially activated at high concentrations of Spd and Spm in tomato fruits overexpressing yeast *SAMDC*, which lead to a stimulation of C sequestration and are associated with higher respiratory rates and higher enzymatic activity for phosphoenolpyruvate carboxylase (PEPC) and NADP-dependent isocitrate dehydrogenase (ICDHc) (Mattoo et al., 2006). Following up on these results, the authors then showed that the ripening-specific accumulation of Spd and Spm in the transgenic tomato fruits results in greater accumulation of glutamine, asparagine, and organic acids in the red fruit and a lower content for valine, aspartate, sucrose, glucose, and choline. Together, the metabolite profile of transgenic tomato fruits suggests the presence of an intricate regulation and interconnections between specific metabolic pathways that are triggered when Spd and Spm reach a specific threshold, which can then act as a signal in the tomato fruit (Mattoo et al., 2007). These findings also suggest that Spm/Spd may serve as a core signal molecule in sugar metabolism and accumulation by N signaling in fruits. The integration of PAs and N signals is related to sugar metabolism and accumulation, which will be a potentially helpful tool to precisely control the rhythm of fruit development.

## Common and Distinct Features of Fruit Ripening in Climacteric Versus Non-Climacteric Fruits

The color break of fruits, which marks the transition from chloroplasts to chromoplasts, is a visual sign for both climacteric and non-climacteric fruits during ripening and is associated with similar changes in sweetness, sourness, texture, and color. Although these processes are controlled by ethylene in climacteric fruits and mainly by ABA in non-climacteric fruits, increasing evidence has uncovered a hidden layer centered on polyamines during ripening. This additional level of control also relies on both common and different mechanisms specific to each fruit type that act at the physiological and molecular levels.

**FIGURE 1:**
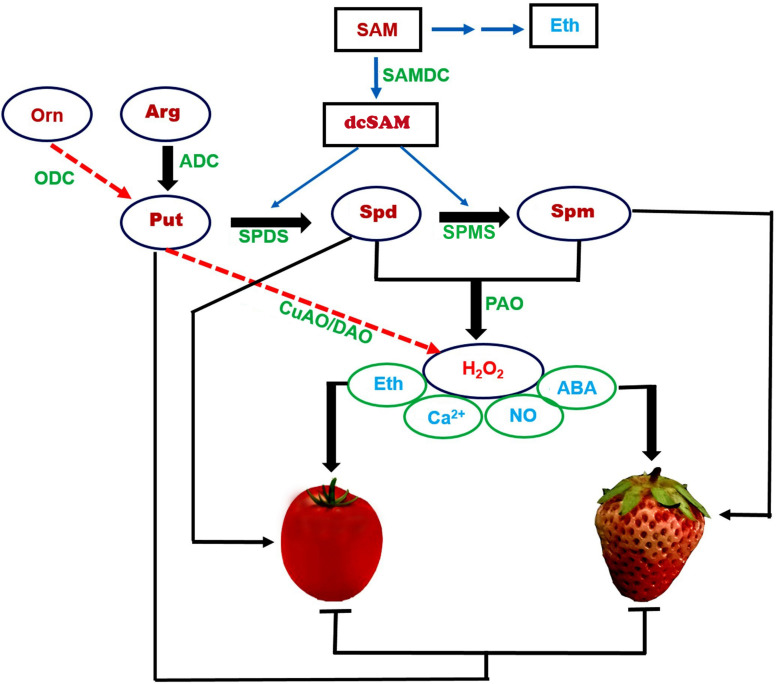
A proposed outline of the roles of polyamines during fruit ripening in climacteric and non-climacteric fruits. We propose a complex regulatory network, centered on the polyamine (PA) compounds Put, Spd, and Spm, metabolic enzymes (ADC, ODC, SAMDC, SPDS, and SPMS), and regulators (ethylene, ABA, NO, H_2_O_2_, and Ca^2+^) in tomato and strawberry, representing climacteric and non-climacteric fruits, respectively. PA biosynthesis begins mainly *via* Arg rather than Orn: ADC is crucial in both climacteric and non-climacteric fruits. SAM-derived dcSAM, catalyzed by SAMDC, is a key step in the biosynthesis of Spd and Spm. High Put levels inhibit fruit ripening, while high Spd and Spm contents promote fruit ripening. A synergetic regulation of SAMDC with SPDS for the production of Spd is critical for climacteric fruits, and of SAMDC with SPMS for the production of Spm for non-climacteric fruits. PAO-mediated PA metabolism of Spd/Spm produces H_2_O_2_ (rather than by CuAO/DAO), which together with ABA, ethylene, NO, and Ca^2+^ constitute a complex network. Put and ethylene form a negatively coordinated loop in climacteric fruit ripening. Spd/Spm and ABA comprise a positively coordinated loop for ripening, especially in non-climacteric fruit ripening. Solid lines represent core metabolic pathways. Dotted lines represent accessory metabolic pathways. The symbols 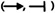 represent promotion and inhibition, respectively. PA, polyamine; Put, putrescine; Spd, spermidine; Spm, spermine; Arg: arginine; Org, ornithine; SAM, *S*-adenosyl-L-methionine; dcSAM, decarboxylated SAM; ODC, ornithine decarboxylase; CuAO/DAO, copper amine oxidase/diamine oxidases; PAO, polyamine oxidases; ADC, arginine decarboxylase; SPDS, Spd synthase; SPMS, Spm synthase; SAMDC, SAM decarboxylase; Eth, ethylene; ABA, abscisic acid; NO, nitric oxide; H_2_O_2_, hydrogen peroxide.

In this context, we review PA compounds (Put, Spd, and Spm), the enzymes involved in their biosynthesis and metabolism (ADC, ODC, SAMDC, SPDS, and SPMS), as well as the relevant phytohormones and regulators (ethylene, ABA, NO, H_2_O_2_, and Ca^2+^), which all constitute a complex regulatory network during fruit development and ripening (summarized in Figure 1). In climacteric fruits, such as tomato, Put and Spd play a dominant role in ripening and senescence. In non-climacteric fruits, such as strawberry, Put and Spm are crucial to ripening and senescence. High Put contents inhibit fruit ripening and senescence, and extend fruit shelf life in both fruit types. While high Spd and Spm contents promote fruit ripening, Spd may dominate in climacteric fruits and Spm in non-climacteric fruits. Broadly speaking, Put works antagonistically to Spd/Spm in cellular metabolism.

Arginine decarboxylase is generally considered to be critical for Put production in both climacteric and non-climacteric fruits. Spd is derived from the catalytic action of SAMDC and SPDS and controls climacteric fruit ripening, while Spm is produced from SAMDC and SPMS and plays a vital role in non-climacteric fruit ripening. PA catabolism generates H_2_O_2_, which integrates a series of signals, mainly ethylene, ABA, NO, and Ca^2+^, thereby accelerating the ripening of both climacteric and non-climacteric fruits. The initiation of Put conversion to Spd/Spm represents a physiological hallmark that corresponds to a transition during fruit developmental processes, mainly associated with the onset of fruit ripening. Conversely, PAO-mediated Spd/Spm metabolism coordinately regulates PA homeostasis and generates a signal, at least in the non-climacteric fruit model plant strawberry.

Because the biosynthesis of both PAs and ethylene employ a common substrate, the balance between PAs and ethylene influences climacteric fruit ripening. By contrast, the interaction between PAs and ABA plays a crucial role in non-climacteric fruit ripening. Based on the available data, we propose a model that describes the role, metabolism, and action mechanisms of PAs during fruit ripening, in the context of climacteric and non-climacteric fruits, at the physiological and molecular levels (Figure 1). PA biosynthesis begins mainly *via* arginine, which is converted to Put by ADC in both climacteric and non-climacteric fruits. Put is then converted to Spd and Spm by the concerted action of SAMDC, SPDS, and SPMS. Decarboxylated SAM (dcSAM), produced by SAMDC from SAM, is a key step in the biosynthesis of Spd and Spm. Thus, a synergetic regulation of SAMDC and SPDS is critical in climacteric fruits for the production of Spd; SAMDC exhibits a similar relationship with SPMS for the production of Spm in non-climacteric fruits. PAO5-catalyzed PA metabolism of Spd/Spm produces H_2_O_2_, which, together with Ca^2+^, triggers crosstalk between multiple signal pathways associated with ABA, ethylene, and NO. Distinct regulatory loops are central to fruit ripening in climacteric and non-climacteric fruits: Put inhibits ethylene production, such that Put and ethylene define a negative feedback loop in ripening, especially in climacteric fruits. Likewise, ABA promotes Spd/Spm biosynthesis, thereby forming a positive feedback loop between Spd/Spm and ABA in ripening, especially in non-climacteric fruits.

In the future, the regulatory mechanism(s) controlling the expression of *ADC*, *SAMDC*, and *PAO5* and their encoded proteins should be examined at the transcriptional and translational levels, respectively. Besides, now that PA transporters have been identified in animal models, including Solute Carrier Family 22 Member 1 (SLC22A1) and multi-drug resistance (MDR1, and ATP-binding cassette protein; Abdulhussein and Wallace, 2014), the search for the PA sensor or receptor has begun. One possibility would be the development of a PA-affinity chromatography technique capable of isolating PA-binding proteins. We suggest that the identification of new components is an important and interesting topic, as they will need to be placed in the context of the existing network among PAs, H_2_O_2_, NO, and Ca^2+^.

## Conclusion

The crucial roles played by PAs in the ripening of both climacteric and non-climacteric fruits depend not only on the composition, contents, and mode(s) of action of PAs but also on the fruit type and developmental stages. Put and Spd/Spm have antagonistic functions, with Put contributing to senescence and Spd/Spm to ripening. ADC is a key enzyme for Put biosynthesis, which prevents ripening of both climacteric and non-climacteric fruits. SAMDC is the key enzyme for the conversion of Put to Spd/Spm, and a rise in its activity coincides with the initiation of ripening, mediated by Spd in climacteric fruits and Spm in non-climacteric fruits. The second messenger H_2_O_2_, derived from PAO activity, facilitates fruit ripening and senescence *via* the coordinated regulation of ABA, ethylene, NO, and Ca^2+^ signaling pathways. Put and ethylene may be considered as a negatively coordinated loop in ripening specifically in climacteric fruits. Their counterpoint in non-climacteric fruits is the positively coordinated loop between Spd/Spm and ABA. At least in strawberry, a signaling molecule derived from PAO5-mediated PA metabolism has been identified that may play a role in non-climacteric fruits.

## Author Contributions

FG, JG, and YS wrote the review. FG and JG collected references. YL and XM designed the model and revised the review. All authors contributed to the article and approved the submitted version.

## Conflict of Interest

The authors declare that the research was conducted in the absence of any commercial or financial relationships that could be construed as a potential conflict of interest.
